# Occlusal characteristics in modern humans with tooth agenesis

**DOI:** 10.1038/s41598-024-56449-9

**Published:** 2024-03-10

**Authors:** Ragda Alamoudi, Georgios Kanavakis, Elias S. Oeschger, Demetrios Halazonetis, Nikolaos Gkantidis

**Affiliations:** 1 Department of Orthodontics and Dentofacial Orthopedics, University of Bern, 3010 Bern, Switzerland; 2 Department of Orthodontics and Pediatric Dentistry, UZB – University School of Dental Medicine, University of Basel, 4056 Basel, Switzerland; 3 Department of Orthodontics, School of Dentistry, National and Kapodistrian University of Athens, 11527 Athens, Greece

**Keywords:** Tooth agenesis, Dentition, Dental occlusion, Malocclusion, Dental overbite, Dental overjet, Anatomy, Oral anatomy

## Abstract

Non-syndromic permanent tooth agenesis affects a significant proportion of the population, especially if third molars are considered. Although tooth agenesis has been linked to a smaller craniofacial size, reduced facial convexity and a shorter skeletal face, the occlusal characteristics of individuals with tooth agenesis remain largely unexplored. Therefore, this study investigated potential associations between tooth agenesis and metric occlusal traits in 806 individuals (491 with 4.1 missing teeth per subject, including third molars, and 315 without any tooth agenesis). Dentoskeletal morphology was defined through anatomical landmarks on pre-treatment cephalometric radiographs. Multivariate regression models, adjusted for sex and age, showed that tooth agenesis was significantly associated with a reduced overjet, an increased interincisal angle, and shorter upper and lower dental arch lengths, but not with overbite. Moreover, apart from reduced tooth length and dentoalveolar effects, as the number of missing teeth increased the upper front teeth were progressively retruded according to the craniofacial complex and to the face. Thus, tooth agenesis has a substantial influence on dental and occlusal characteristics, as well as on the sagittal position and inclination of anterior teeth. These findings emphasize the necessity for personalized, multidisciplinary approaches in individuals with multiple agenesis to successfully meet treatment goals.

## Introduction

Non syndromic tooth agenesis comprises a common congenital dental anomaly, evident in about 6.4% of the population^1,2^, without considering the third molars. Ancestry and sex have an impact on the prevalence of this dental anomaly, with females showing a higher risk compared to males^1^. Moreover, isolated agenesis of at least one third molar has been reported in 22.6% of the Caucasian population. Here, females are also more affected with a 14% higher prevalence of third molar agenesis compared to males, and the maxilla is more often affected than the mandible^3–5^. Despite differences in prevalence, recent reports did not detect any sex discrepancies in the patterns of tooth agenesis when all teeth were investigated, including the third molars^6^. Third molar agenesis shows a higher prevalence in the Asian population (29.7%), while the lowest rate is observed in African populations (5.7%)^3,7^.

Tooth agenesis is related to genetic or epigenetic factors that are also involved in overall craniofacial development^8–10^. From an evolutionary viewpoint, it is argued that humans have experienced a reduction in tooth size and number as a response to a reduction in functional needs^11,12^. This evolutionary mechanism appears to be active in modern humans, influencing the number of teeth, craniofacial size, and craniofacial shape in a coordinated manner^13–15^. Isolated third molar agenesis has also been associated with craniofacial size and shape, with the effects being more pronounced compared to the agenesis of other teeth^14,16^.

Phenotypically, individuals with tooth agenesis present a less convex craniofacial complex, as well as a shorter lower facial third. These morphological differences become more notable as the severity of tooth agenesis increases and are equally pronounced in males and females^15,16^. More specifically, tooth agenesis has been shown to result in a more retruded maxilla, protruded mandible, shorter anterior facial height, and a reduced skeletal profile convexity. The presence of such craniofacial differences between individuals with missing teeth and individuals with a full permanent dentition, allows for speculation regarding the presence of analogous effects on occlusal characteristics, such as overjet and overbite or the position of the incisors relative to the jaws and the face.

The correlation between dental occlusion and tooth agenesis has been rarely studied, with publications using Angle classification of malocclusion, which focuses on the position of the first molars^17^. Angle Class I occlusion is considered normal, with the upper first molar’s mesio-buccal cusp fitting between the lower molars’ buccal cusps. In Class II malocclusion, the upper molar’s mesio-buccal cusp is located more posteriorly, while in Class III it is located more anteriorly compared to Class I. Meta-analytical data from five studies^18–22^ with significant geographic variations, have indicated a higher prevalence of tooth agenesis in Class III malocclusion compared to Class I or Class II, with an odds ratio of 2.15 (95% CI 0.78–5.89), without considering third molars^1^. These findings should be treated with caution since geographic variations could confound malocclusion prevalence, as well as agenesis patterns. Consistent with this meta-analysis, a recent study reported a 16.2% tooth agenesis prevalence in Class III malocclusion compared to 2.2% in Class I and 3.6% in Class II malocclusion subgroups^23^. Class II division 2 malocclusion, characterized by reduced overjet, despite the Angle Class II dental relation, has also been associated with an increased prevalence of tooth agenesis in certain studies^24,25^, although these findings were not corroborated by other research^19,26^.

The aforementioned research findings could suggest a potential link between tooth agenesis and features of dental occlusion. However, the prevalence of Class III and Class II division 2 malocclusion types, which showed differences in the incidence of tooth agenesis, is limited in the population and largely linked to genetic predisposition^19,26–28^. All assessments thus far have been conducted on stratified groups based on the Angle classification—a somewhat ambiguous categorization that lacks a basis in biological principles. The Angle classification is a qualitative assessment focusing primarily on the relationships of the first molars, as perceived by Edward Angle^17^, disregarding several other occlusal features, such as the position and angulation of the upper front teeth relative to each other, as well as to the face^29^. Additionally, in case of tooth agenesis, the present teeth tend to drift, usually mesially, affecting molar relationship at the respective side, and thus, confounding the Angle classification.

Therefore, the primary outcome of the present study was to investigate potential associations between tooth agenesis and metric occlusal traits in a large sample of modern humans, selected consecutively without considering any malocclusion traits. Further, unlike previous reports, third molars were also included in the assessment.

## Material and methods

### Ethical approval

The protocol for this observational case control study was reviewed and approved by the Ethics Commission of the Canton of Bern, Switzerland (Project-ID: 2018-01340), and the Research Committee of the School of Dentistry, National and Kapodistrian University of Athens, Greece (Project-ID: 281, 9 February 2016). The study was conducted in accordance with the Declaration of Helsinki. For reporting, the STROBE criteria were followed. Participants whose information was used in the study and/or their legal guardians provided written informed consent.

### Sample

This study is part of a larger project studying tooth agenesis characteristics and potential associations with the craniofacial form^4–6,13–16^. The sample used in the present study is almost identical to the one described in detail in a previous publication^15^. Only necessary information to understand the current manuscript will be reported here. The study population was derived from consecutive orthodontic patient records archived between 2002 and December 2017, at the following orthodontic clinics: (a) University of Bern, Switzerland; (b) National and Kapodistrian University of Athens, Greece; (c) two private practices in Athens and two in Thessaloniki, Greece; and (d) one private practice in Biel, Switzerland.

Inclusion criteria:

1. Permanent tooth agenesis (congenitally missing) including the third molars.
2. No systemic diseases, craniofacial malformations, syndromes, or any other anomalies affecting craniofacial morphology, as reported in the subjects’ medical records.
3. European (White) ancestry.
4. Individuals older than 8 years of age and younger than 40 years of age.
5. Lateral cephalometric radiograph in maximal intercuspation of adequate clinical diagnostic quality and with a reference ruler at the mid-sagittal level.
6. Panoramic radiographs of adequate diagnostic quality.
7. No history of interventions known to influence craniofacial morphology, such as orthodontic treatment.
8. Absence of any other severe dental anomaly regarding tooth number, size, or form in any tooth except from third molars.
9. Individuals for whom the reason for any missing tooth was known. Panoramic radiographs obtained at an age older than 12 years were retrieved from all individuals younger than 12 years old at the time of the pre-treatment radiographs^30,31^.

Data collection was performed by reviewing the medical and dental history, the clinical photographs and the radiographs of each individual, and all relevant data were then recorded in an Excel sheet (Version 2312, Microsoft Excel, Microsoft Corporation, Redmond, WA, USA, https://www.microsoft.com/en-us/microsoft-365/excel). The TAC (Tooth Agenesis Code) system was used to record the tooth agenesis patterns. It employs a binary arithmetic to characterize the presence or absence of each tooth, providing a unique value for each pattern^5,32^. Third molar agenesis was also recorded, but the presence of third molars was not taken into account during sample collection.

From a total of 808 individuals, 402 individuals with permanent tooth agenesis (238 females, 164 males; Median age 13.0 years, range: 8.0–38.3 years; mean: 2.2 missing teeth per subject) and 404 control individuals without tooth agenesis, not considering third molars, were included in the present study. The control individuals were matched to the initial agenesis sample for age (within 6 months), sex, and geographic origin. Two male patients were excluded from the agenesis group due to impacted or missing upper incisors that did not allow for outcome assessment^15^. The current sample is almost identical to previously published study populations^4,5,13,15^. In the present study third molar agenesis was considered in the sample. Therefore, the final sample (n = 806) consisted of 491 individuals with tooth agenesis, including third molars (mean: 4.1 missing teeth per subject) and 315 individuals without any tooth agenesis. Detailed age and sex distribution according to chronological age has been reported previously and showed a balanced sample (Supplementary Fig. 1)^15^.

### Radiograph digitization and measured outcomes

**Figure 1:**
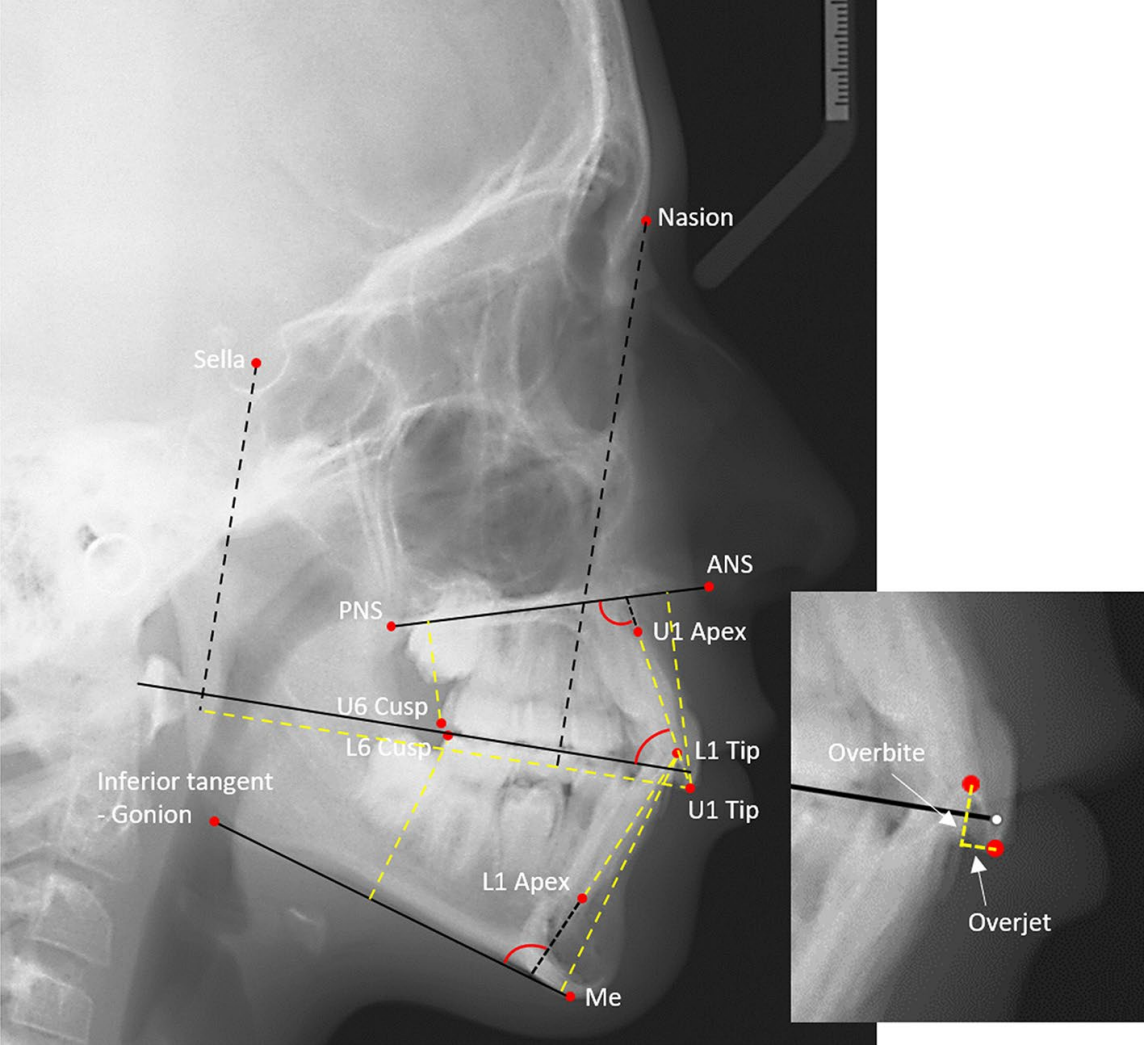
Cephalometric image depicting the twelve fixed landmarks (red circles), the planes (black lines), as well as the linear (yellow lines) and angular (red curves) measurements performed in the study.

All pre-treatment lateral cephalometric digital images were uploaded on Viewbox 4 software (version 4.1.0.12 BETA, dHAL software, Kifissia, Greece, http://www.dhal.com/viewbox.htm) for digitization and were scaled to real size, using a reference ruler depicted in the radiographs. One trained operator positioned twelve fixed skeletal and dental anatomical landmarks on each cephalometric image (Fig. 1). The definition of the used dental landmarks is provided in Supplementary Table 1.

The primary and secondary study outcomes are reported in Table 1. All data were exported from Viewbox 4 software in an Excel sheet (Microsoft Excel, Microsoft Corporation, Redmond WA, USA), including information about age, sex, date of birth, date of image acquisition and specific tooth agenesis patterns.

**Table 1:** Definition of the study outcomes.

Occlusal traits (primary outcomes)	
Interincisal angle (°)	The inner angle measured between the long axis of U1 and L1
Overjet (mm)	Distance of U1 to L1 along the functional occlusal plane
Overbite (mm)	Distance of U1 to L1 along a line perpendicular to the functional occlusal plane
Upper functional dental arch length (mm)	Distance of U1 tip to U6 point
Lower functional dental arch length (mm)	Distance of L1 tip to L6 point
Dentoalveolar variables (secondary outcomes)	
U1 to palatal plane (°)	The inner angle formed by the U1 and the palatal plane
U1 to palatal plane (mm)	The vertical distance of U1 incisal tip to palatal plane
U6 to palatal plane (mm)	The vertical distance of U6 point to palatal plane
L1 to mand. plane (°)	The inner angle between the long axis of L1 and the mandibular plane
L1 to mand. plane (mm)	The vertical distance of L1 incisal tip to mandibular plane
L6 to mand. plane (mm)	The vertical distance of L6 point to mandibular plane
Dentoskeletal variables (secondary outcomes)	
Sagittal position of U1 to craniofacial complex (mm)	The perpendicular distance of U1 incisal tip from a line passing through Sella and vertical to occlusal plane
Vertical position of U1 to craniofacial complex (mm)	The perpendicular distance of U1 incisal tip from a line passing through Sella and parallel to occlusal plane
Sagittal position of U1 to face (mm)	The perpendicular distance of U1 incisal tip from a line passing through Nasion and vertical to occlusal plane
Dental variables (secondary outcomes)	
U1 length (mm)	The linear distance between the incisal tip and the root apex of the maxillary central incisor (following the root canal, if visible). The most anteriorly positioned lateral incisor was used in case of agenesis
L1 length (mm)	The linear distance between the incisal tip and the root apex of the mandibular central incisor (following the root canal, if visible). The most anteriorly positioned lateral incisor was used in case of agenesis

The effect of number of missing teeth on each illustrated group of variables was tested through multivariate analysis.

Abbreviations, landmarks, and planes are described in Supplementary Table 1.

### Statistical analysis

The statistical analysis was conducted with IBM SPSS Statistics for Windows (Version 29.0. Armonk, NY: IBM Corp, https://www.ibm.com/spss). A two-sided significance test was carried out at an alpha level of 0.05. A Bonferroni correction was applied on the level of statistical significance, where required.

Data were tested for normality through the Kolmogorov–Smirnov test and visualization of data distribution histograms, Q–Q and P–P plots and no important deviations were detected. Equality of variances was checked through Levene´s test and equality of covariance matrices through Box’s test. No significant assumption test violations were noticed, and therefore, following preliminary and exploratory testing, four multivariate regression models were applied to the data (general linear models) to test for the effect of number of missing teeth (including third molars) on the: (a) metric occlusal traits (5 dependent variables: interincisal angle, overjet, overbite, upper, and lower functional dental arch length), (b) dentoalveolar traits (6 dependent variables: U1 to palatal plane angle and distance, U6 to palatal plane distance, L1 to mandibular plane angle and distance, and L6 to mandibular plane distance), (c) dentoskeletal traits (3 dependent variables: sagittal and vertical position of U1 to craniofacial complex, and sagittal position of U1 to face), and (d), dental traits (2 dependent variables: U1 and L1 tooth length). All models were adjusted for the effects of sex (fixed factor) and age (covariate) factors. The first model was used for the primary and the rest three models used for the secondary study outcomes (Table 1). The decision to create separate models for the aforementioned dependent variables was based on anatomical and statistical considerations. This approach was chosen to minimize the risk of false positive effects that can arise when conducting multiple post-hoc statistical tests within a single, large multivariate model. Observed * predicted * standardized residual plots were visualized to verify the suitability of the applied models and revealed a good fit across the whole range of data in all cases.

Landmark identification was repeated in 30 randomly selected radiographs, one month after the initial process, to test for the intra-operator error in the measured variables. A Wilcoxon signed rank-test was used to assess systematic error and the average and standard deviation of the absolute differences between repeated measurements was indicative of the random error.

## Results

### Method error

There was no systematic error in any of the measured variables (p > 0.003, Bonferroni correction applied) and the random error was also negligible. The highest random error was detected for interincisal angle at 0.91 ± 0.93°, which is considered acceptable.

### Association of metric occlusal traits to number of missing teeth

**Figure 2:**
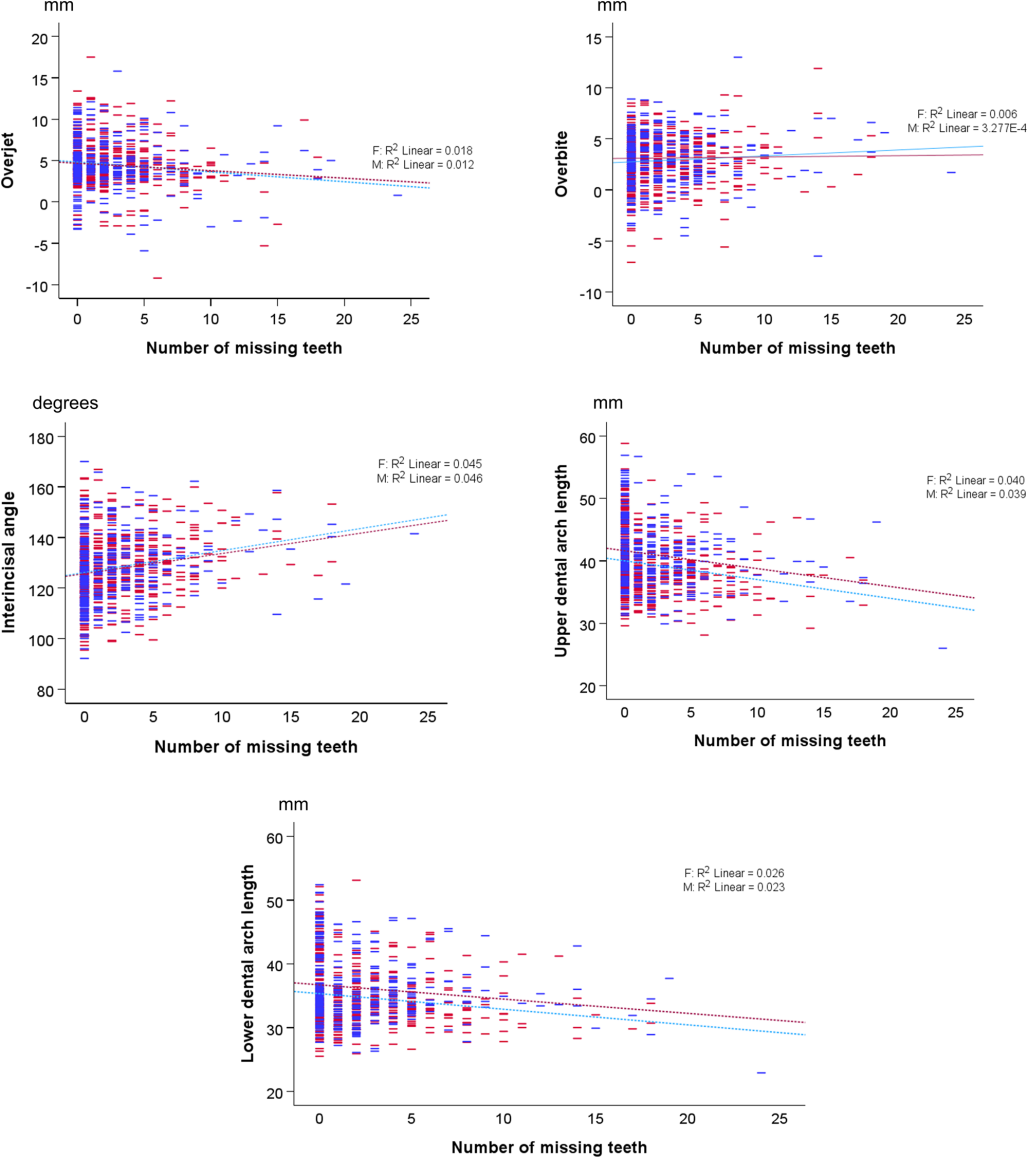
Scatter plots showing the association of the five occlusal trait variables to the number of missing teeth in males (blue) and females (red). The dashed lines represent the linear regression lines fitted to each group.

After controlling for age and sex, multivariate testing showed that the number of missing teeth had a significant effect on occlusal traits (P < 0.001) (Table 2). The effects on each occlusal trait variable are shown in Table 3 and in Fig. 2.

**Table 2:** Results of multivariate regression analysis testing the effects of age, number of missing teeth, and sex on metric occlusal traits, dentoalveolar, dentoskeletal, and dental variables (n = 331 males and 477 females).

Factors	Partial eta squared	P-value*
Dependent variables: occlusal traits (overjet, overbite, interincisal angle, upper dental arch length, lower dental arch length)		
Age	0.034	< 0.001
Number of missing teeth	0.064	< 0.001
Sex	0.045	< 0.001
Dependent variables: dentolalveolar variables (U1 to palatal plane distance, U6 to palatal plane distance, L1 to mandibular plane angle and distance, L6 to mandibular plane distance)		
Age	0.167	< 0.001
Number of missing teeth	0.097	< 0.001
Sex	0.095	< 0.001
Dependent variables: dentoskeletal variables (sagittal and vertical position of U1 to craniofacial complex, sagittal position of U1 to face)		
Age	0.187	< 0.001
Number of missing teeth	0.075	< 0.001
Sex	0.150	< 0.001
Dependent variables: dental variables (U1 length, L1 length)		
Age	0.022	< 0.001
Number of missing teeth	0.041	< 0.001
Sex	0.074	< 0.001

*Wilks' Lambda Test.

**Table 3:** Parameter estimates indicating the effect of tested factors on each occlusal trait variable (dependent variable).

Dependent variable	Parameter	β coefficient	95% confidence interval		P-value
			Lower bound	Upper bound	
Overjet	Intercept	5.10	4.49	5.71	< 0.001
	Age	− 0.02	− 0.05	0.01	0.134
	Number of missing teeth	− 0.10	− 0.16	− 0.04	0.001
	Female (Ref.: male)	0.07	− 0.33	0.47	0.727
Overbite	Intercept	2.63	2.10	3.17	< 0.001
	Age	0.02	0.01	0.05	0.080
	Number of missing teeth	0.03	− 0.02	0.08	0.256
	Female (Ref.: male)	− 0.25	− 0.59	0.10	0.161
Interincisal angle	Intercept	123.93	121.19	126.68	< 0.001
	Age	0.10	− 0.04	0.23	0.151
	Number of missing teeth	0.80	0.54	1.07	< 0.001
	Female (Ref.: male)	0.59	− 1.20	2.37	0.519
Dental arch length upper	Intercept	40.28	39.24	41.31	< 0.001
	Age	0.08	0.03	0.13	0.002
	Number of missing teeth	− 0.32	− 0.42	− 0.22	< 0.001
	Female (Ref.: male)	− 1.70	− 2.37	− 1.03	< 0.001
Dental arch length lower	Intercept	34.95	33.90	35.99	< 0.001
	Age	0.10	0.05	0.15	< 0.001
	Number of missing teeth	− 0.27	− 0.37	− 0.16	< 0.001
	Female (Ref.: male)	− 1.49	− 2.17	− 0.81	< 0.001

The number of missing teeth showed a significant association with overjet, interincisal angle, upper arch length, and lower arch length (P ≤ 0.001). Specifically, as the number of missing teeth increased, overjet decreased by 0.10 mm per tooth unit (95% CI − 0.16 to − 0.04), while the interincisal angle increased by 0.80° per tooth unit (95% CI 0.54 to 1.07). Additionally, both upper and lower dental arch lengths decreased by 0.32 and 0.27 mm per tooth unit, respectively, with an increasing number of missing teeth (95% CI − 0.42 to − 0.22; − 0.37 to − 0.16, respectively). However, the association between the number of missing teeth and overbite was not statistically significant (P > 0.05) (Table 3).

The estimated marginal means for these occlusal trait variables were tested between sex groups and statistically significant differences were found only for upper and lower dental arch length (P < 0.001; Table 4).

**Table 4:** Marginal estimates for metric occlusal traits in the different sex groups.

Dependent variable	Sex	Mean^a^	Std. error	95% confidence interval		P-value
				Lower bound	Upper bound	
Overjet	F	4.5	0.1	4.3	4.8	0.727
	M	4.5	0.2	4.1	4.8	
Overbite	F	2.9	0.1	2.7	3.1	0.161
	M	3.1	0.1	2.9	3.4	
Interincisal angle	F	128.3	0.6	127.1	129.4	0.519
	M	127.7	0.7	126.3	129.1	
Dental arch length upper	F	39.2	0.2	38.8	39.7	< 0.001
	M	40.9	0.3	40.4	41.4	
Dental arch length lower	F	34.7	0.2	34.3	35.1	< 0.001
	M	36.2	0.3	35.7	36.7	

^a^Covariates appearing in the model are evaluated at the following values: Age = 18.2 years, Number of missing teeth = 2.47.

### Association of dentoalveolar variables to number of missing teeth

**Figure 3:**
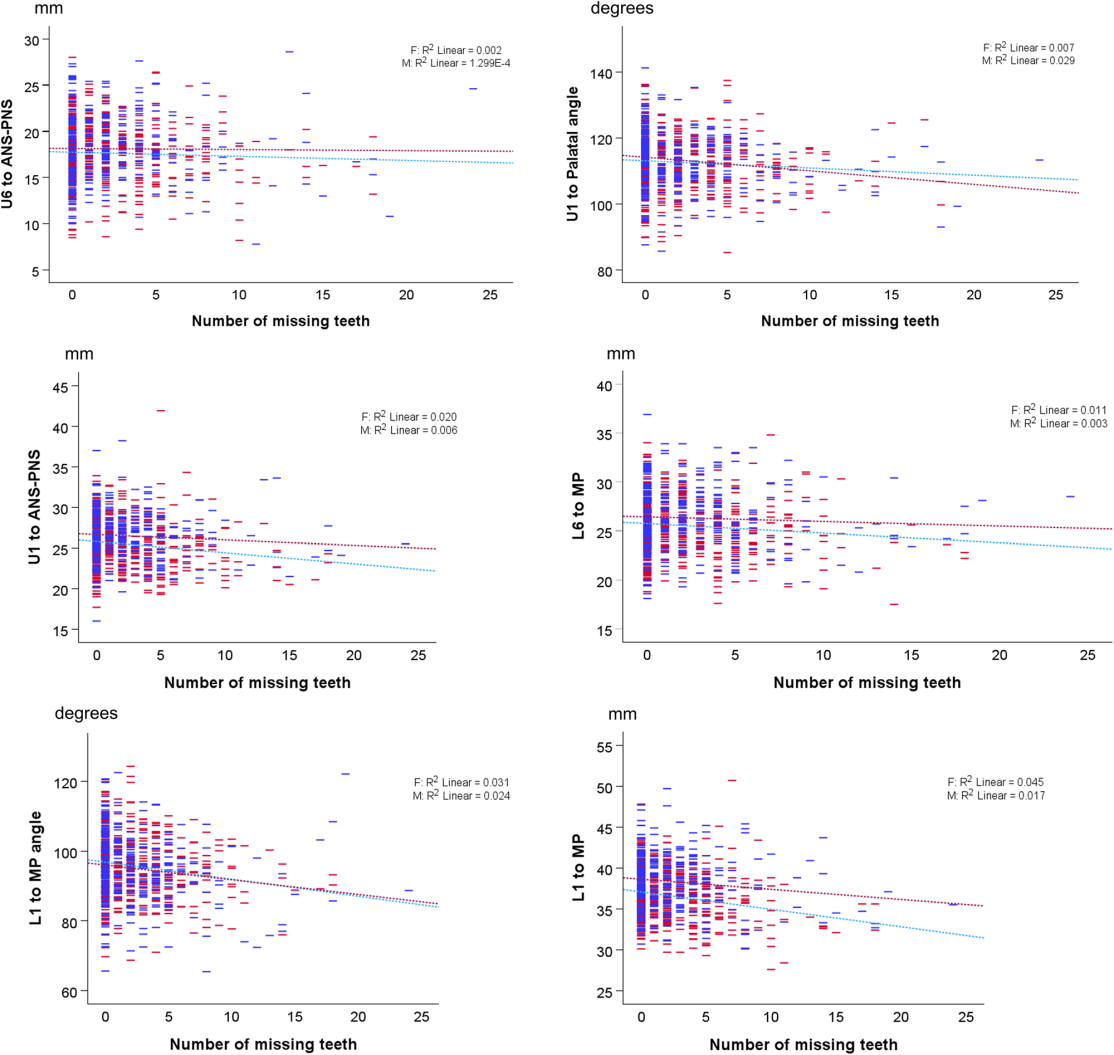
Scatter plots showing the association of the six dentoalveolar variables to the number of missing teeth in males (blue) and females (red). The dashed lines represent the linear regression lines fitted to each group.

After controlling for age and sex, multivariate testing showed that the number of missing teeth also had a statistically significant effect on dentoalveolar variables (P < 0.001) (Table 2). The effects on each dentoalveolar variable are shown in Supplementary Table 2 and in Fig. 3.

The number of missing teeth shows a significant association with all tested dentoalveolar variables (P < 0.003). Parameter testing showed that U1 to palatal plane angle decreases by 0.29° for every tooth that is missing (95% CI − 0.46 to − 0.11) and U1 to palatal plane distance decreases by 0.14 mm per missing tooth (95% CI − 0.20 to − 0.08). Additionally, U6 to palatal plane distance decreases by 0.08 mm (95% CI − 0.15 to − 0.01), and L6 to mandibular plane distance decreases by 0.12 mm for every missing tooth (95% CI − 0.18 to − 0.06). Finally, L1 to mandibular plane angle and L1 to mandibular plane distance decrease by 0.49° and 0.22 mm per missing tooth, respectively (95% CI − 0.68 to − 0.30; − 0.28 to − 0.16) (Supplementary Table 2). Supplementary Table 3 reports the marginal estimates for the different sex groups.

### Association of dentoskeletal variables to number of missing teeth

**Figure 4:**
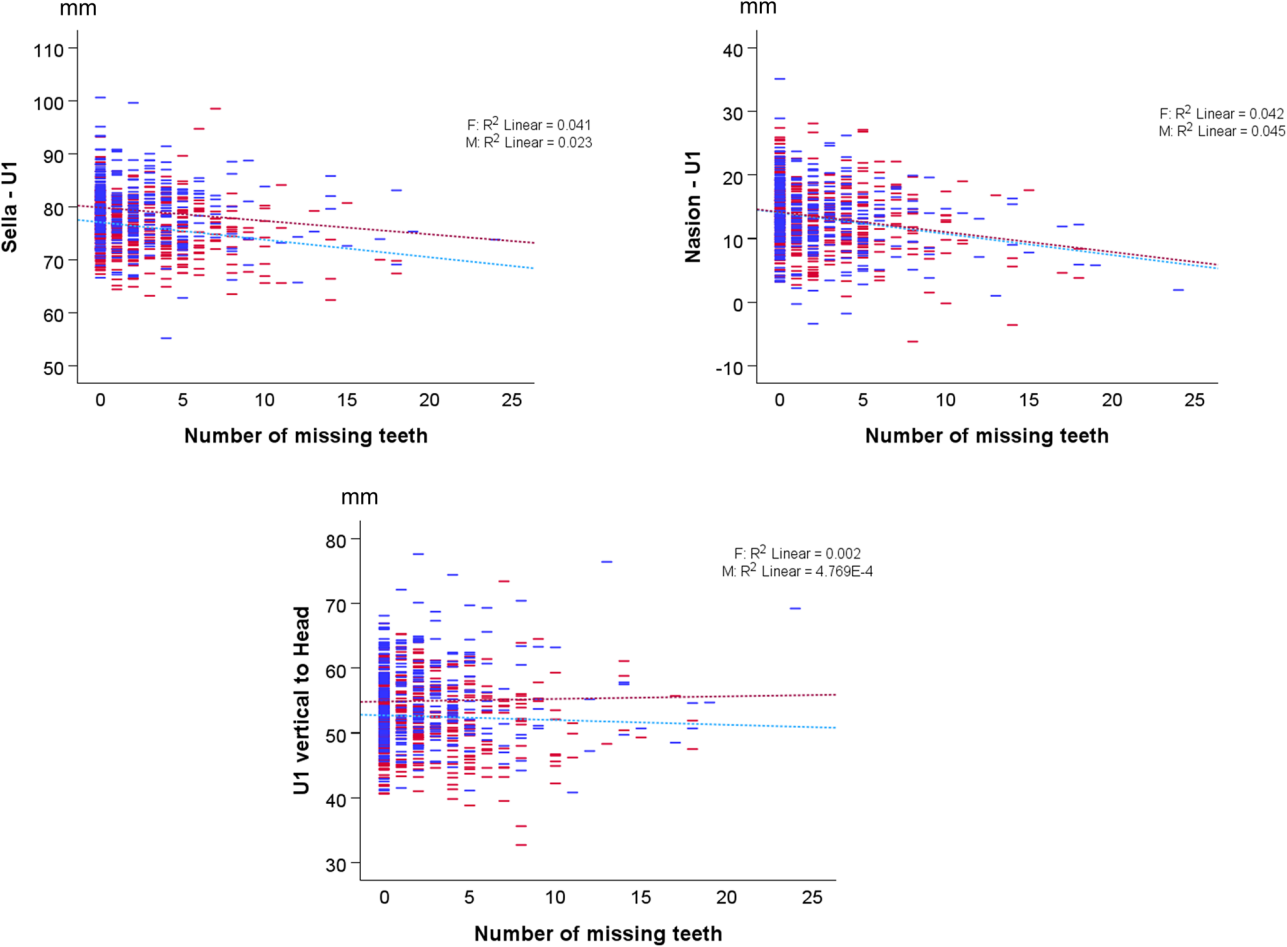
Scatter plots showing the association of the three dentoskeletal variables to the number of missing teeth in males (blue) and females (red). The dashed lines represent the linear regression lines fitted to each group.

Significant results were evident when the number of missing teeth was regressed against dentoskeletal variables, after controlling for age and sex (P < 0.001) (Table 2). The specific effects on each dentoskeletal variable are shown in Supplementary Table 4 and in Fig. 4.

The number of missing teeth showed a significant association with the sagittal position of U1 to craniofacial complex and to the face (P < 0.001). Specifically, as the number of missing teeth increased, the sagittal position of U1 to craniofacial complex decreased by 0.35 mm per missing tooth (95% CI − 0.45 to  − 0.24) and the sagittal position of U1 to the face decreased by 0.32 mm per missing tooth (95% CI − 0.43 to − 0.22). There was no association between the number of missing teeth and the vertical position of U1 to craniofacial complex (P > 0.05) (Supplementary Table 4). Supplementary Table 5 reports the marginal estimates for the different sex groups.

### Association of dental variables to number of missing teeth

There was a statistically significant association between the number of missing teeth and dental variables, after controlling for age and sex (P < 0.001) (Table 2). The specific effects on each dental variable are shown in Supplementary Table 6 and Supplementary Fig. 2.

The number of missing teeth was significantly associated with the upper and lower anterior tooth length (P < 0.001). Specifically, as the number of missing teeth increased, the U1 length decreased by 0.09 mm per missing tooth (95% CI − 0.13 to − 0.04) and the L1 length decreased by 0.14 mm per missing tooth (95% CI − 0.18 to − 0.09) (Supplementary Table 6). Supplementary Table 7 reports the marginal estimates for the different sex groups.

## Discussion

The present study investigated the association between non-syndromic tooth agenesis and metric occlusal traits, dentoalveolar variables, dentoskeletal characteristics, as well as anterior tooth length in a large sample. Tooth agenesis is the most prevalent congenital dental anomaly in the population, impacting a little less than one-third of individuals, when third molars are taken into consideration^1,2^. In this study, the third molars were included in the analyses, as third molar agenesis relates to the agenesis of other teeth^4^ and also has a more profound impact on human craniofacial morphology, than the agenesis of other teeth^14,16^. The significance of this study lies in increasing our understanding for the relationship between tooth agenesis and dentofacial features and thereby adds valuable insight in the exploration of the mechanisms involved in dental and craniofacial development^11,12^.

The results show that the number of missing teeth is associated with various occlusal traits. Specifically, as the number of missing teeth increased, there was an incremental decrease in overjet, an increase in the interincisal angle, and a reduction in both upper and lower dental arch lengths. On the other hand, no significant association was found between the number of missing teeth and overbite. Similar results were also found for the corresponding sagittal, dentoalveolar, and dentoskeletal variables examined in this study. Specifically, an increased number of missing teeth was linked to a reduction in the upper incisor to palatal plane angle and the lower incisor to mandibular plane angle. Additionally, a more retruded sagittal position of the upper incisors was observed relative to both the craniofacial complex and the anterior facial structures. These findings are supported by recent reports regarding the interincisal angle, the overjet, and the incisor inclinations in individuals with maxillary lateral incisor agenesis^33^. Hence, tooth agenesis appears to affect the sagittal position of the incisors, and subsequently impacts sagittal occlusal traits, with potential impact on facial esthetics^34–36^. These results are in accordance to those of previous studies using Angle classification, which showed a clear association of tooth agenesis to Class III malocclusion^1,18–22^. On the other hand, our findings do not indicate a clear association with Class II div. 2 malocclusion, perhaps apart from the increased interincisal angle. In any case, outcome interpretation using Angle classification should be performed with caution for reasons reported in the introduction.

In the vertical dimension, the position of teeth in relation to their skeletal bases was also affected by the number of missing teeth. Vertical distances between the upper and lower incisors and first molars to their respective skeletal bases showed significant reduction as the number of missing teeth increased. A reduction in the vertical distance of the upper central incisor from the palatal plane has been reported in the literature in adults with maxillary lateral incisors agenesis^37^. However, overbite values remained unaffected perhaps due to the reduced incisor length in individuals with tooth agenesis. A reduction in the dentoalveolar and dentoskeletal linear variables might be attributed to the effects of tooth agenesis on craniofacial morphology, namely the smaller facial size and the shorter anterior facial height^13–16,38^. Associations between vertical facial patterns and variations in overjet and overbite have been also shown in individuals with complete permanent dentition^39^. These associations, however, do not appear to have an influence on the soft tissue profile at least in middle aged individuals, with sagittal relationships having a stronger impact^36,40^.

Sexual dimorphism was investigated in all multivariate models and its effects were statistically significant. However, post-hoc analysis revealed that despite differences between sexes in certain variables and small differences in magnitudes of effects, both sexes were similarly affected by tooth agenesis. In accordance with previous studies, the upper and lower dental arch lengths were significantly smaller in females compared to males^41,42^. The same was true for the length of the central incisors. This finding was anticipated, as females have smaller tooth crowns^43^ and shorter roots than males^44^. The vertical position of U1 to palatal plane, the vertical distance of L6 to mandibular plane as well as the sagittal and vertical distances of U1 to the craniofacial complex were also reduced in females, which was expected due to the smaller craniofacial size compared to males. Thus, despite the potential difficulties associated with evaluating certain dental parameters on cephalometric radiographs, mainly due to the overlapping of various anatomical structures in the apical region, the results affirmed the precision of the measurements.

The present study found a significant association between the number of missing teeth and several dentoalveolar characteristics. As the number of missing teeth increased, the vertical position of the upper and lower incisors relative to the palatal plane or mandibular plane, as well as the inclination of the upper incisors to the palatal plane, decreased. This suggests that tooth agenesis can have a negative impact on dental and dentofacial aesthetics, as well as on smile attractiveness, not only due to missing teeth per se, but also through the effects on incisor position and angulation^45–47^. For instance, the sagittal position of the upper incisor in relation to the face is strongly related to upper lip position^48^, and thus an increased retral angulation of the upper incisors will probably lead to a more retro positioned upper lip. The incisor length was also reduced in individuals with tooth agenesis. Previous studies also found smaller teeth crowns with differences increasing with agenesis severity^49,50^. If the dental effects surpass the skeletal effects in size reduction, this might have an additional negative impact on dental esthetics.

Furthermore, as the number of missing teeth increased, the upper incisors were more retropositioned relative to the craniofacial complex and face. These findings are in accordance with the craniofacial morphology patterns detected previously in individuals with tooth agenesis^15^; namely, a more retruded maxilla, a more protruded mandible, and an overall reduced craniofacial convexity. Due to the reduced proclination of upper anterior teeth in individuals with tooth agenesis the dental effects are exceeding beyond the skeletal effects, with potentially important implications for esthetics^51^.

The argument that the dental effects exceed the skeletal effects is also supported by the association of the tested variables with the number of missing third molars, investigated on a subsample including exclusively individuals with all other teeth present (females: 237, males: 167). These analyses showed that after controlling for age and sex, the number of missing third molars did not have a significant effect on occlusal traits (P = 0.215), as well as on dentoalveolar variables (P = 0.285) (Supplementary text, Supplementary Table 8, Supplementary Fig. 3). When considering that the third molar agenesis has a more profound impact on craniofacial morphology than the agenesis of other teeth^16^, along with the present findings, it becomes evident that the detected impact of tooth agenesis on occlusal traits can be attributed partially to localized dental effects. These might occur as part of a dental compensation mechanism in response to missing teeth within the dental arches.

The aforementioned findings, whether they pertain to overall craniofacial morphology or more specific local effects, align with the human evolutionary trend of decreasing facial size and convexity^11,12^. At present, it is uncertain whether the occlusal characteristics observed in individuals with tooth agenesis primarily result from effects in their skeletal configurations or if they are also directly affected by the evolutionary shift towards fewer and smaller number and size of teeth, along with reduced facial dimensions^13–16^.

The robustness of the present results is strengthened by the large sample size and the inclusion of third molars, which are often overlooked in similar studies, despite their significant association to tooth agenesis and craniofacial morphology^4,14,16^. In addition, the use of multivariate regression models allowed for the assessment of multiple dependent variables while controlling for potential confounding factors, such as age and sex. The study population consisted of well documented individuals whose development was evaluated for a certain period of time, enhancing the diagnostic ability and reducing the chances of misdiagnosis. However, certain limitations should be considered when interpreting the results. The sample population was restricted to individuals with white-European ancestry, limiting the generalizability of the findings to other populations^1,52^. Also, the analyzed lateral cephalometric radiographs provide valuable information, but have inherent limitations in capturing three-dimensional craniofacial features^53^.

## Conclusion

The outcomes of this study suggest that after controlling for age and sex, tooth agenesis has a significant impact on various dental and metric occlusal traits. An increased number of missing teeth was found to be associated with reduced overjet, increased interincisal angle, and shorter upper and lower dental arch lengths, but not with overbite. Tooth agenesis also had a notable influence on sagittal dentoalveolar and dentoskeletal variables, resulting in a more retruded sagittal position and reduced labial inclination of the anterior teeth. Addressing these challenges is particularly crucial when treating orthodontic patients with multiple teeth agenesis, highlighting the need for personalized, multidisciplinary treatment approaches for such individuals.

The observed associations contribute to our understanding of the broader implications of tooth agenesis. It not only affects craniofacial morphology, as part of an evolutionary trend towards reduced facial size and number of teeth, but also extends its influence to encompass dental and occlusal features. This has strong clinical implications and perhaps also developmental and evolutionary implications.

### Supplementary Information

Supplementary Information.

## Data Availability

The data presented in this study are available on request from the corresponding author.
