# Supplementary material for: Occlusal characteristics in modern humans with tooth agenesis
Source: Sci Rep. 2024 Mar 10;14:5840. doi: 10.1038/s41598-024-56449-9 (PMC10925589; doi:10.1038/s41598-024-56449-9)
Supplement: Supplementary file 1 — Supplementary Information. [file 41598_2024_56449_MOESM1_ESM.pdf]

Supplementary material - Scientific Reports

Occlusal characteristics in modern humans with tooth agenesis

Ragda Alamoudi, Georgios Kanavakis, Elias S. Oeschger, Demetrios Halazonetis, and Nikolaos Gkantidis

Supplementary Table 1. Definition of the digitized dental landmarks and the planes used in the study.

Skeletal landmarks	
Nasion	The most anterior point of the frontonasal suture in the midsagittal plane
Sella	The midpoint of the pituitary fossa (sella turcica)
Dental landmarks	
U1	The most frontal central incisor tip: if missing, the most frontal tooth was used.
L1	The most frontal lower central incisor tip: if missing, the most frontal tooth was used.
U6	The most distal part of the occlusal surface of the most distal upper molar that was fully erupted; in case of double contours, the middle was digitized, even if thus considered one second and one third molar.
L6	The most distal part of the occlusal surface of the most distal lower molar that was fully erupted; in case of double contours, the middle was digitized, even if thus considered one second and one third molar.
Planes	
Mandibular plane	A plane tangent to the lower border of the mandible defined anteriorly from the point Me and posteriorly from the inferior tangent-Gonion point.
Palatal plane	A plane defined anteriorly from ANS and posteriorly from PNS.
Functional occlusal plane	A plane defined anteriorly by a point bisecting the overbite and posteriorly by bisecting the occlusal curve formed by the upper and lower occluded premolars and molars.

Supplementary table 2. Parameter estimates indicating the effect of tested factors on each dentoalveolar variable (dependent variable).

Dependent Variable	Parameter	β coefficient	95% CI		p-Value	Partial Eta Squared
			Lower Bound	Upper Bound		
U6 to ANS-PNS (mm)	Intercept	15.38	14.68	16.08	<0.001	0.700
	Age	0.16	0.13	0.20	<0.001	0.101
	Number of missing teeth	-0.08	-0.15	-0.01	0.028	0.006
	(Ref.: male)	-0.57	-1.03	-0.12	0.013	0.008
U1 to Palatal plane angle (°)	Intercept	115.22	113.41	117.02	<0.001	0.951
	Age	-0.08	-0.17	0.01	0.080	0.004
	Number of missing teeth	-0.29	-0.46	-0.11	0.002	0.012
	(Ref.: male)	-0.55	-1.73	0.62	0.358	0.001
U1 to ANS-PNS (mm)	Intercept	24.40	23.79	25.01	<0.001	0.883
	Age	0.14	0.11	0.17	<0.001	0.092
	Number of missing teeth	-0.14	-0.20	-0.08	<0.001	0.027
	(Ref.: male)	-1.11	-1.51	-0.71	<0.001	0.036
L6 to MP (mm)	Intercept	23.84	23.22	24.45	<0.001	0.879
	Age	0.15	0.12	0.18	<0.001	0.115
	Number of missing teeth	-0.12	-0.18	-0.06	<0.001	0.019
	(Ref.: male)	-0.89	-1.29	-0.49	<0.001	0.023
L1 to MP angle (°)	Intercept	94.44	92.51	96.38	<0.001	0.920
	Age	0.10	0.01	0.19	0.045	0.005
	Number of missing teeth	-0.49	-0.67	-0.30	<0.001	0.031
	(Ref.: male)	0.62	-0.64	1.87	0.337	0.001
L1 to MP (mm)	Intercept	35.91	35.26	36.56	<0.001	0.937
	Age	0.17	0.13	0.20	<0.001	0.118
	Number of missing teeth	-0.22	-0.28	-0.16	<0.001	0.055
	(Ref.: male)	-1.86	-2.28	-1.44	<0.001	0.086

Supplementary table 3. Estimated marginal means for dentoalveolar variables in the different sex groups.

Dependent Variable	Sex	Mean[a]	Std. Error	95% CI		P-value
				Lower Bound	Upper Bound	
U6 to ANS-PNS (mm)	F	17.59	0.15	17.30	17.88	0.013
	M	18.17	0.18	17.82	18.51	
U1 to Palatal plane (°)	F	112.54	0.38	111.79	113.29	0.358
	M	113.09	0.46	112.19	113.99	
U1 to ANS-PNS (mm)	F	25.42	0.13	25.17	25.68	<0.001
	M	26.53	0.16	26.23	26.84	
L6 to MP (mm)	F	25.47	0.13	25.21	25.72	<0.001
	M	26.35	0.16	26.05	26.66	
L1 to MP (°)	F	95.60	0.41	94.80	96.41	0.337
	M	94.98	0.49	94.02	95.95	

a. Covariates appearing in the model are evaluated at the following values: Age = 18.2, Number of missing teeth = 2.47.

Supplementary table 4. Parameter estimates indicating the effect of tested factors on each dentoskeletal variable (dependent variable).

Dependent Variable	Parameter	β coefficient	95% CI		P-Value	Partial Eta Squared
			Lower Bound	Upper Bound		
Sella - U1 (mm)	Intercept	76.93	75.80	78.07	<0.001	0.957
	Age	0.18	0.12	0.23	<0.001	0.047
	Number of missing teeth	-0.35	-0.46	-0.23	<0.001	0.044
	(Ref.: male)	-3.08	-3.81	-2.34	<0.001	0.077
Nasion - U1 (mm)	Intercept	14.18	13.09	15.28	<0.001	0.448
	Age	0.01	-0.05	0.05	0.990	0.000
	Number of missing teeth	-0.32	-0.43	-0.22	<0.001	0.042
	(Ref.: male)	-0.16	-0.87	0.55	0.664	0.000
U1 vertical to Head (mm)	Intercept	49.15	47.97	50.34	<0.001	0.892
	Age	0.34	0.28	0.40	<0.001	0.144
	Number of missing teeth	-0.12	-0.24	0.01	0.043	0.005
	(Ref.: male)	-2.60	-3.38	-1.83	<0.001	0.052

Supplementary table 5. Estimated marginal means for dentoskeletal variables in the different sex groups.

Dependent Variable	Sex	Mean[a]	Std. Error	95% CI		P-value
				Lower Bound	Upper Bound	
Sella - U1 (mm)	F	76.21	0.24	75.74	76.68	<0.001
	M	79.28	0.29	78.72	79.85	
Nasion - U1 (mm)	F	13.22	0.23	12.77	13.68	0.664
	M	13.38	0.28	12.83	13.92	
U1 vertical to Head (mm)	F	52.46	0.25	51.96	52.95	<0.001
	M	55.06	0.30	54.47	55.65	

a. Covariates appearing in the model are evaluated at the following values: Age = 18.2, Number of missing teeth = 2.47.

Supplementary table 6. Parameter estimates indicating the effect of tested factors on each dental variable (dependent variable).

Dependent Variable	Parameter	β coefficient	95% CI		P-Value	Partial Eta Squared
			Lower Bound	Upper Bound		
U1 length (mm)	Intercept	24.88	24.43	25.32	<0.001	0.937
	Age	0.04	0.02	0.06	<0.001	0.018
	Number of missing teeth	-0.09	-0.13	-0.04	<0.001	0.018
	(Ref.: male)	-1.17	-1.46	-0.87	<0.001	0.071
L1 length (mm)	Intercept	23.04	22.57	23.51	<0.001	0.921
	Age	0.01	-0.02	0.03	0.510	0.001
	Number of missing teeth	-0.14	-0.18	-0.09	<0.001	0.041
	(Ref.: male)	-0.88	-1.18	-0.58	<0.001	0.039

Supplementary table 7. Estimated marginal means for dental variables in the different sex groups.

Dependent Variable	Sex	Mean[a]	Std. Error	95% CI		P-value
				Lower Bound	Upper Bound	
U1 length (mm)	F	24.27	0.10	24.08	24.45	<0.001
	M	25.43	0.11	25.21	25.66	
L1 length (mm)	F	21.96	0.10	21.77	22.16	<0.001
	M	22.84	0.12	22.61	23.07	

a. Covariates appearing in the model are evaluated at the following values: Age = 18.2, Number of missing teeth = 2.47

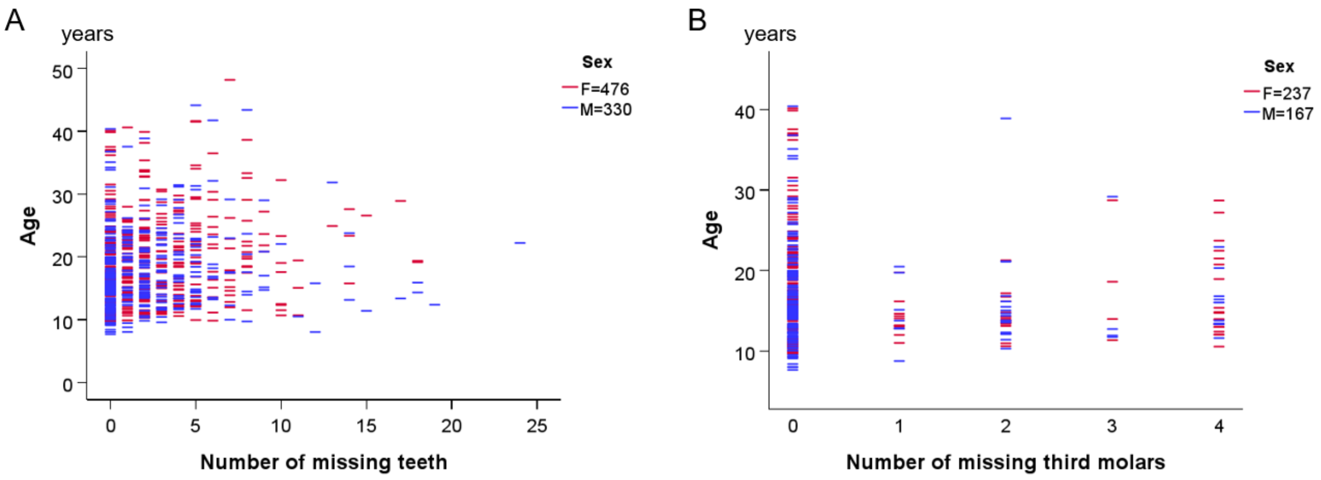

Supplementary figure 1. A. Sexual distribution of number of missing teeth per age in the entire sample, **B.** Sexual distribution of number of missing third molars per age in individuals without tooth agenesis in other teeth.

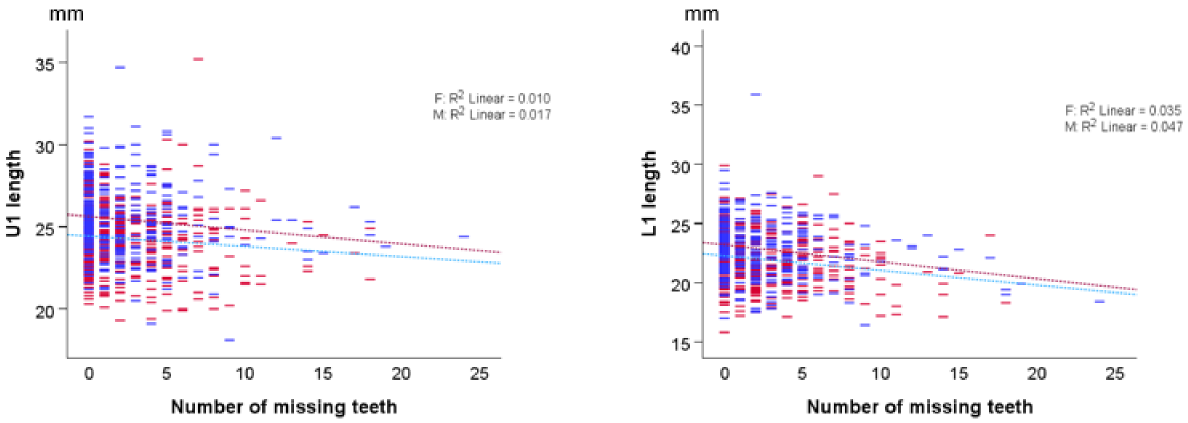

Supplementary figure 2. Scatter plots showing the association of both dental variables to the number of missing teeth in males (blue) and females (red). The dashed lines represent the linear regression lines fitted to each group.

Association of occlusal traits, dentoalveolar, dentoskeletal, and dental variables to number of missing third molars

To test for these outcomes, similar multivariate models to that applied in the entire study sample were applied on a subsample including exclusively individuals with all teeth other than third molars formed (F = 237, M = 167). Misdiagnosis of later formed third molars in the younger individuals of this sample was partially controlled by the inspection of available radiographs obtained at an older age and by the inclusion of the age factor in these models. After controlling for age and sex, the multivariate testing showed that the number of missing third molars did not have a significant effect on occlusal traits (P = 0.215), as well as on dentoalveolar variables (P = 0.285). On the contrary, significant results were evident when the number of missing third molars was regressed against

dentoskeletal variables (P = 0.006), as well as against dental variables (P < 0.001) (Supplementary table 7).

The number of missing third molars showed a significant association with the sagittal position of U1 to craniofacial complex and to the face (P = 0.003). Specifically, as the number of missing third molars increased, the sagittal position of U1 to craniofacial complex decreased by 0.65 mm per missing tooth (95% CI =-1.08 to -0.23). There was no association between the number of missing teeth and the sagittal position of U1 to the face (P = 0.075) or the vertical position of U1 to the craniofacial complex (P = 0.091). Regarding the dental variables, as the number of missing third molars increased, the U1 length decreased by 0.39 mm per missing tooth (95% CI = -0.55 to -0.23) and the L1 length decreased by 0.45 mm per missing tooth (95% CI = -0.61 to -0.29) (Supplementary figure 1).

Supplementary table 8. Results of multivariate regression analysis testing the effects of age, number of missing third molars, and sex on occlusal traits, dentoalveolar, dentoskeletal, and dental variables (n = 167 males and 237 females).

Dependent variables (Occlusal traits): Overjet, Overbite, Interincisal angle, Upper dental arch length, Lower dental arch length		
Factors	Partial Eta Squared	P-Value*
Age	0.174	<0.001
Number of missing teeth	0.018	0.215
Sex	0.053	<0.001
Dependent variables (Dentolalveolar variables): U1 to palatal plane distance, U6 to palatal plane distance, L1 to mandibular plane angle and distance, L6 to mandibular plane distance		
Factors	Partial Eta Squared	P-Value*
Age	0.120	<0.001
Number of missing teeth	0.018	0.285
Sex	0.091	<0.001
Dependent variables (Dentoskeletal variables): sagittal and vertical position of U1 to craniofacial complex, sagittal position of U1 to face		
Factors	Partial Eta Squared	P-Value*
Age	0.119	<0.001
Number of missing teeth	0.031	0.006
Sex	0.125	<0.001
Dependent variables (Dental variables): U1 length, L1 length		
Factors	Partial Eta Squared	P-Value*
Age	0.071	<0.001
Number of missing teeth	0.084	<0.001
Sex	0.117	<0.001

*Wilks' Lambda Test

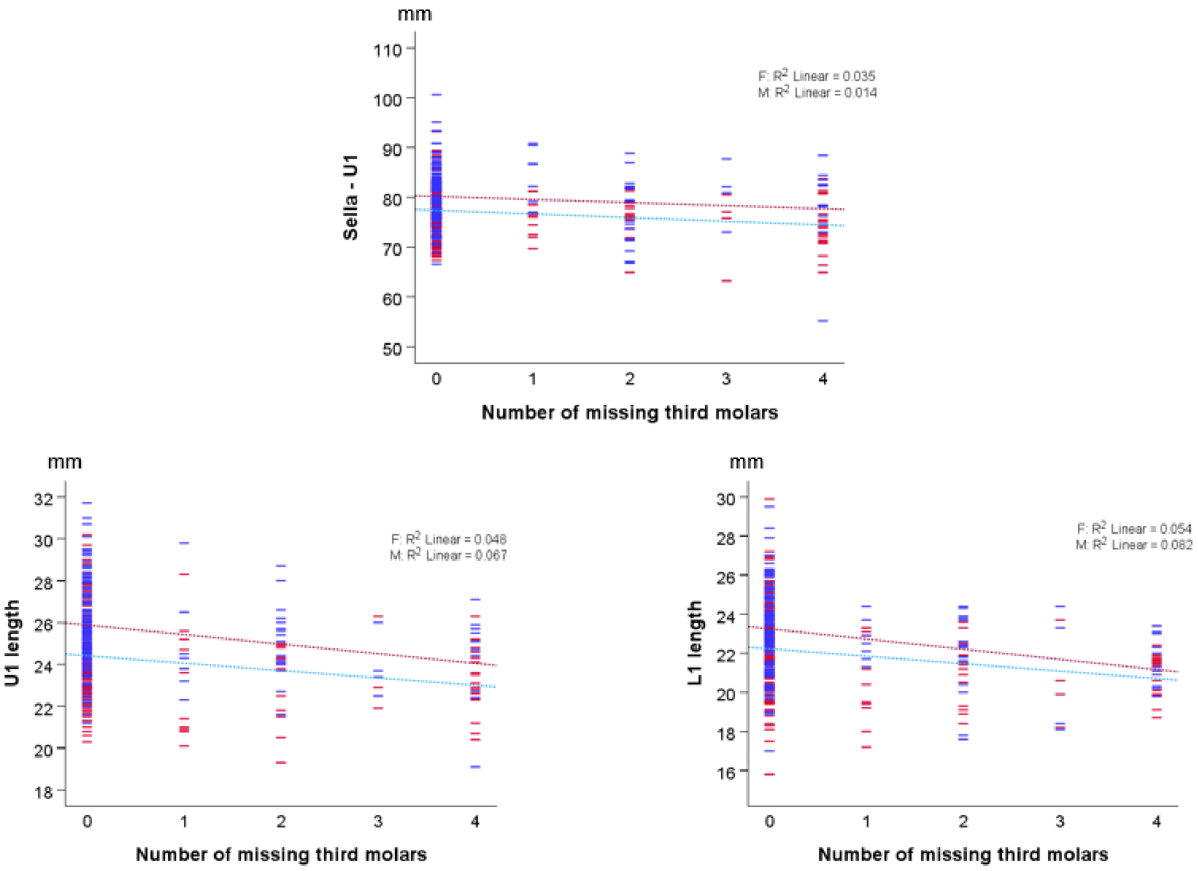

Supplementary figure 3. Scatter plots showing the significant associations between one dentoskeletal (upper image: sagittal position of the upper central incisor to craniofacial complex) and two dental variables (upper and lower incisor tooth length) to the number of missing third molars per sexual group. These data concern individuals with all teeth other than third molars formed (F = 237, M = 167). The dashed lines represent the linear regression lines fitted to each group.
